# Supplementary material for: Higher order gaps in the renormalized band structure of doubly aligned hBN/bilayer graphene moiré superlattice
Source: Nat Commun. 2024 Mar 14;15:2335. doi: 10.1038/s41467-024-46672-3 (PMC10940307; doi:10.1038/s41467-024-46672-3)
Supplement: Supplementary file 1 — Supplementary Information [file 41467_2024_46672_MOESM1_ESM.pdf]

Supplementary Information
Higher order gaps in the renormalized band structure of doubly aligned hBN/bilayer graphene moiré superlattice

Mohit Kumar Jat[1†], Priya Tiwari[2†], Robin Bajaj[1†], Ishita Shitut[1], Shinjan Mandal[1], Kenji Watanabe[3], Takashi Taniguchi[4], H. R. Krishnamurthy[1], Manish Jain[1,*] and Aveek Bid[1†]

[1]*Department of Physics, Indian Institute of Science, Bangalore 560012, India*

[2]*Braun Center for Submicron Research, Department of Condensed Matter Physics, Weizmann Institute of Science, Rehovot, Israel*

[3] *Research Center for Electronic and Optical Materials, National Institute for Materials Science, 1-1 Namiki, Tsukuba 305-0044, Japan*

[4] *Research Center for Materials Nanoarchitectonics, National Institute for Materials Science, 1-1 Namiki, Tsukuba 305-0044, Japan*

[†] *These authors contributed equally.*

CONTENTS

Supplementary Note 1: Device fabrication 2

Supplementary Note 2: Determining the relative twist between BLG and hBN 3

A: Device $\mathrm{D_{single}}$ - single moiré device 3

B: Device $\mathrm{D_{double}}$ - supermoiré device 3

C: Size of the supermoiré cell 4

Supplementary Note 3: Determining stacking type 4

Supplementary Note 4: Continuum Hamiltonian 5

A: Model Description 5

B: Band Structure Calculation 6

C: Density of states (DOS) versus number density 6

Supplementary Note 5: Determining the Bragg indices for the gaps 7

Supplementary Note 6: Quasi Brillouin Zones 7

Supplementary Note 7: Comparison of qBZ in SLG and BLG 8

Supplementary Note 8: Analysis of previously published data on SLG supermoiré 10

Supplementary Note 9: Landau fan diagram 12

Supplementary Note 10: Absence of supermoiré peak in resistance measurements 12

Supplementary Note 11: Comparing hBN double moiré system of SLG and BLG 12

Supplementary References 15

Supplementary References 15

SUPPLEMENTARY NOTE 1: DEVICE FABRICATION

We fabricated bilayer graphene (BLG) heterostructures doubly aligned with the single crystalline hBN of thickness $20 - 25$ nm. The flakes were mechanically exfoliated on Si/SiO_2 wafers. The number of layers in the graphene flake was determined from Raman spectroscopy (Supplementary Figure 1). AFM was used to measure the thicknesses of the hBN flakes and ensure their uniformity.

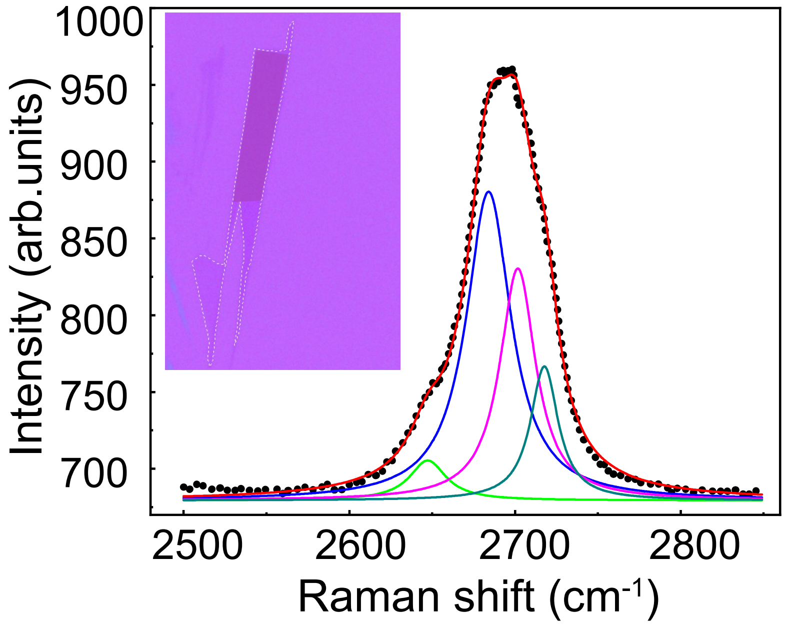

Supplementary Figure 1. Raman spectra. A plot of the 2D Raman spectra of the bilayer graphene. The black-filled circles are the experimentally measured Raman data. The red solid line is cumulative of the four Lorentzian fitted to it; the four Lorentzian are also individually shown. The inset shows an optical image of the BLG flake; the shaded region is the part of the BLG used for device fabrication.

The constituent layers of the heterostructure were sequentially transferred on each other using a dry transfer technique (Supplementary Figure 2). During the transfer, the edges of the crystals were carefully aligned (aiming for less than 1° misalignment) to form the moiré heterostructure. In the first step, a BLG flake was aligned to an hBN flake at a near-zero angle such that a moiré superstructure forms between the entire top surface of BLG and the hBN (Supplementary Figure 2(d)). Half the BLG flake was then covered with a single-layer WSe_2 (Supplementary Figure 2(e)). An hBN flake was then picked up by the stack, ensuring that it had a near-zero angular mismatch with the BLG (Supplementary Figure 2(f)). The two halves of the BLG were then separated into two different devices, labelled $\mathrm{D_{double}}$ and $\mathrm{D_{single}}$, respectively (Supplementary Figure 2(h)). The BLG in device $\mathrm{D_{double}}$ formed a double moiré heterostructure with the top- and the bottom-hBN flakes. Device $\mathrm{D_{single}}$, on the other hand, formed a moiré heterostructure with only the top-hBN. The results presented in the main manuscript are from device $\mathrm{D_{double}}$. The device $\mathrm{D_{single}}$ (which is fabricated from the same BLG and top hBN flake as device $\mathrm{D_{double}}$) forms the ideal control device for comparison of the characteristics of single- and double-moiré heterostructures of hBN and BLG.

Electron beam lithography was used to pattern electrical contacts, followed by reactive ion etching (using a mixture CHF_3 (40 sscm) + O_2 (4 sscm)) and thermal deposition of Cr/Au (5nm/55nm) to create one-dimensional edge contacts [1]. The devices were then etched to define the Hall bar geometry. In the last step, top gates were fabricated using electron beam lithography and thermal deposition of Cr/Au (5nm/55nm). The presence of dual-gated device architecture provides the freedom to tune the charge carrier density n and displacement field D independently via the relations $n = (C_{tg}V_{tg} + C_{bg}V_{bg})/e + n_0$ and $D = (C_{bg}V_{bg} - C_{tg}V_{tg})/2 + D_0$ (the effective electric field in the system is D/ϵ_0). Here n_0 is the residual charge density due

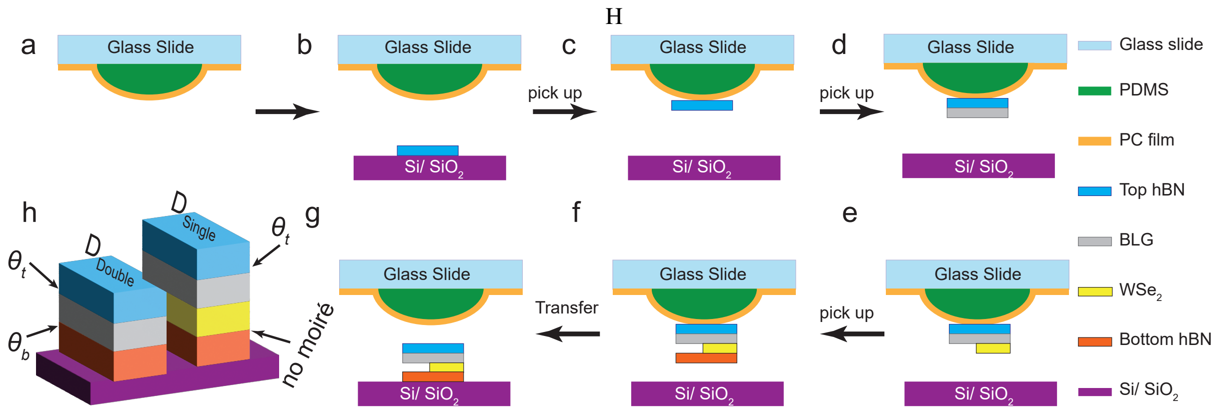

Supplementary Figure 2. Device fabrication schematic. (a) PDMS dome covered with a thin film of PC. (b) Desired top hBN flake is brought in contact with the PC at 120°C. (c) After successful pickup, Si/SiO_2 wafer is brought down. (d) A BLG flake is picked up. (e) A WSe_2 flake is picked up on one-half of the BLG. (f) The bottom hBN flake is picked. (g) The heterostructure is transferred on clean Si/SiO_2 substrate by melting PC at 190°C. (h) The heterostructure is finally etched in two halves.

to doping, and D_0 is the net internal displacement field. C_{tg} and C_{bg} are the top and bottom gate capacitance respectively; their values are extracted from quantum hall measurements.

SUPPLEMENTARY NOTE 2: DETERMINING THE RELATIVE TWIST BETWEEN BLG AND HBN

A: Device $\mathrm{D_{single}}$ - single moiré device

Supplementary Figure 3(a) shows a plot of the longitudinal resistance R_{xx} versus carrier density n measured for the single-moiré device $\mathrm{D_{single}}$. The resistance peak at $n_t = \pm 2.8 \times 10^{16}\ \mathrm{m}^{-2}$ is a consequence of the opening of a moiré gap (MG) at this number density. The moiré wavelength was calculated using the relation:

$$\lambda^2 = \frac{8}{\sqrt{3}n} \qquad \text{(Supplementary Equation 1)}$$

to be $\lambda_t = 12.84\ \mathrm{nm}$. The twist angle between the BLG and the top hBN was estimated using the general relation between twist angle and the moiré wavelength [2–4]:

$$\lambda = \frac{(1+\epsilon)\mathrm{a}}{[\epsilon^2 + 2(1+\epsilon)(1-\cos(\theta))]^{1/2}} \qquad \text{(Supplementary Equation 2)}$$

Here $\mathrm{a} = 0.246\ \mathrm{nm}$ is the lattice constant of graphene, $\epsilon = 0.018$ is the lattice mismatch between the hBN and graphene, and θ is the relative rotational angle between the two lattices. We find the magnitude of the twist angle between the BLG and the top hBN to be $|\theta_t| = 0.44°$.

B: Device $\mathrm{D_{double}}$ - supermoiré device

The plot of R_{xx} versus n for the device $\mathrm{D_{double}}$ shows split moiré gaps (SMG) at $n_b = \pm 2.36 \times 10^{16}\ \mathrm{m}^{-2}$ and $n_t = \pm 2.80 \times 10^{16}\ \mathrm{m}^{-2}$ (Supplementary Figure 3(b)). This indicates an alignment of the BLG with both the top and bottom hBN layers. From the positions of the SMG, we extract the two moiré wavelengths to be $\lambda_b = 13.96\ \mathrm{nm}$ and $\lambda_t = 12.84\ \mathrm{nm}$ (Supplementary Equation 1). The magnitudes of the corresponding twist angles are $|\theta_b| = 0.03°$ and $|\theta_t| = 0.44°$ (Supplementary Equation 2). Recalling that (1) these two devices have common BLG and top hBN flakes and (2) the top hBN forms a moiré with twist angle $|\theta_t| = 0.44°$ with the BLG (as seen in the previous section from the data for device $\mathrm{D_{single}}$); we conclude that the moiré formed between the bottom hBN and BLG has a moiré wavelength $\lambda_b = 13.96$ nm and a twist angle $|\theta_b| = 0.03°$.

We find the frequencies of the Brown-Zak oscillations to be $f_b = 24.5$ T ($\lambda_b = 13.97$ nm) and $f_a = 29$ T ($\lambda_t = 12.84$ nm) (see main manuscript). The corresponding carrier densities which satisfy the conditions for BZ oscillations are $|n_b| = 2.36 \times$

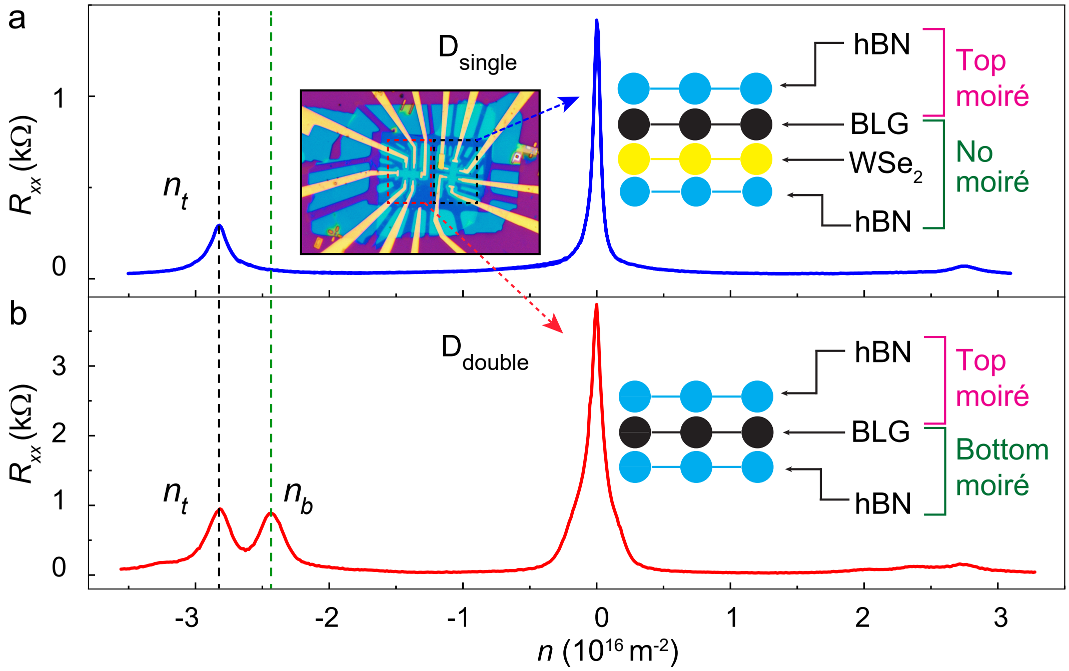

Supplementary Figure 3. Longitudinal resistance comparison for single and supermoiré. (a) Plot of longitudinal resistance R_{xx} versus n for the single moiré device D_{single}. The inset shows the schematic of the layer stacking. The resistance peak at $n_t = -2.8 \times 10^{16}$ m^{-2} (shown by the dashed black line) is from the moiré reconstruction of electronic bands. (b) Plot of R_{xx} versus n for the supermoiré device D_{double}. The inset shows the schematic of the layer stacking. Two secondary resistance peaks are seen at $n_b = -2.36 \times 10^{16}$ m^{-2} (green dashed line) and $n_t = -2.8 \times 10^{16}$ m^{-2} (black dashed line).

10^{16} m^{-2} and $|n_t| = 2.8 \times 10^{16}$ m^{-2}. These values match extremely well with the locations of the secondary moiré peaks (Supplementary Figure 3(b)). In the device, the calculated impurity carrier density is ($\delta n_i \approx 7 \times 10^{14}$ m^{-2}), which gives an uncertainty in twist angle estimation to be $\delta\theta \approx 0.03°$.

C: Size of the supermoiré cell

The number density at which supermoiré gap opens is given analytically by Ref. [5]:

$$n_s = -\frac{16[cos(\theta_b - \theta_t) - 1]}{\sqrt{3}a^2(1+\delta)^2}. \qquad \text{(Supplementary Equation 3)}$$

Using Supplementary Equation 3, we find that for θ_b and θ_t in opposite direction, the supermoiré gap should open at a number density 0.5×10^{16} m^{-2}. On the other hand, for θ_b and θ_t in the same relative direction, the supermoiré gap should open at a number density 0.38×10^{16} m^{-2}. The second value matches very well with our experimentally identified gap location of $n_s = 0.39 \times 10^{16}$ m^{-2}. We thus conclude that the hBN at the top and the bottom have the same direction of the relative twist angles with the intervening BLG. We summarize the observations of this section in the Supplementary Table 1:

Interface	number density (n)	moiré wavelength (λ)	relative twist angle (θ)
Bottom hBN and BLG	$n_b = 2.36 \times 10^{16}$ m^{-2}	$\lambda_b = 13.97$ nm	$\theta_b = 0.03° \pm 0.03°$
Top hBN and BLG	$n_t = 2.80 \times 10^{16}$ m^{-2}	$\lambda_t = 12.84$ nm	$\theta_t = 0.44° \pm 0.03°$

Supplementary Table 1. Calculated moiré wavelengths, number density at SDP, and moiré angles at the two interfaces of the BLG with hBN.

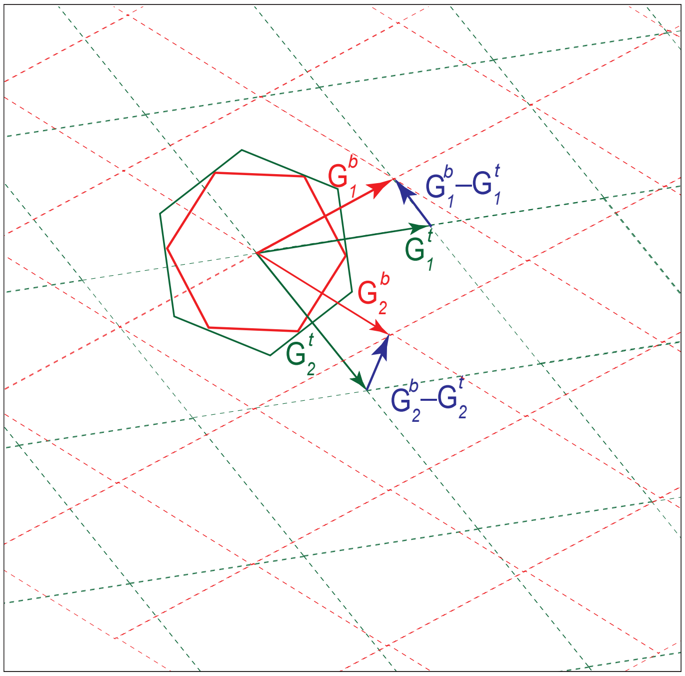

Supplementary Figure 4. A schematic showing the supermoiré reciprocal lattice vectors $\mathbf{G}_1^b - \mathbf{G}_1^t$ and $\mathbf{G}_2^b - \mathbf{G}_2^t$.

SUPPLEMENTARY NOTE 3: DETERMINING STACKING TYPE

There are five possible stacking configurations of the hBN/BLG/hBN heterostructure (Supplementary Figure 5) [6]. We find that Bragg gaps appear at the primary Dirac point and the secondary Dirac points only for the AB1 stacking. Although the nature of stacking is experimentally unidentified, the agreement of the AB1 stacking calculations and experiments indicates that B and N stacking is of AB1 type.

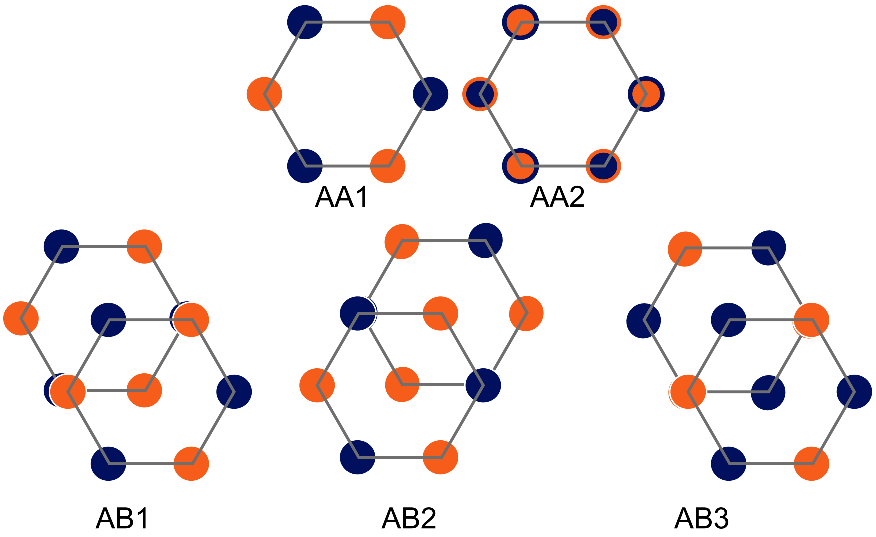

Supplementary Figure 5. Schematic of the five stacking arrangements of hBN layers [6]. The B and N atoms are shown in orange and blue, respectively.

SUPPLEMENTARY NOTE 4: CONTINUUM HAMILTONIAN

A: Model Description

The higher order fractal gaps observed in the experiments can be theoretically understood by calculating the energy spectrum using the effective continuum model for hBN/BLG/hBN system with the twist angles, θ_t and θ_b for the top and bottom hBN/graphene respectively. In general, generic pairs of θ_b and θ_t leads to two incommensurate moiré periods. We construct the commensurate approximant close to the experimentally observed twist angles and write the effective Hamiltonian. For this quadrilayer system, we write the 8×8 Bistritzer-MacDonald Continuum Hamiltonian in the sublattice basis $\{A^b, B^b, A^1, B^1, A^2, B^2, A^t, B^t\}$ as

$$H = \begin{bmatrix} H_{hBN} & U^b & 0 & 0 \\ U^{b\dagger} & H_G & U^\dagger_{BLG} & 0 \\ 0 & U_{BLG} & H_G & U^{t\dagger} \\ 0 & 0 & U^t & H_{hBN} \end{bmatrix} \qquad \text{(Supplementary Equation 4)}$$

where $A^b, B^b, A^1, B^1, A^2, B^2, A^t, B^t$ are sublattice sites of bottom hBN, two graphene layers, and top hBN respectively. The BLG is AB stacked such that A^1 and B^2 are vertically aligned. H_G and H_{hBN} are the Hamiltonian of graphene and hBN. The off-diagonal blocks, U^b, U^t and U_{BLG} are the interlayer hybridization matrix elements for top hBN/graphene, bottom hBN/graphene and bilayer graphene, respectively.

We reduce the 8×8 Hamiltonian to 4×4 effective Hamiltonian, eliminating the sublattice basis of hBN using second-order perturbation and writing it as:

$$H_{eff} = \begin{bmatrix} H_G + V^b_{hBN} & U^\dagger_{BLG} \\ U_{BLG} & H_G + V^t_{hBN} \end{bmatrix} \qquad \text{(Supplementary Equation 5)}$$

where in the low-energy limit, $V^\ell_{hBN} = U^{\ell\dagger}(-H_{hBN})^{-1}U^\ell = v_0 + v_1 e^{\iota\xi \mathbf{G}^\ell_1\cdot\mathbf{r}} + v_2 e^{\iota\xi \mathbf{G}^\ell_2\cdot\mathbf{r}} + v_3 e^{\iota\xi \mathbf{G}^\ell_3\cdot\mathbf{r}}$ with $\ell = b, t$. ξ is the valley index. $\mathbf{G}^\ell_1$ and $\mathbf{G}^\ell_2$ are reciprocal lattice basis vectors of the ℓ moiré and $\mathbf{G}^\ell_3 = -\mathbf{G}^\ell_1 - \mathbf{G}^\ell_2$. The twist angle dependence is reflected in the $\mathbf{G}^\ell_{1,2}$ in the potential.

v_0, v_1, v_2 and v_4 are given by $V_0 \begin{bmatrix} 1 & 0 \\ 0 & 1 \end{bmatrix}$, $V_1 e^{\iota\xi\phi} \begin{bmatrix} 1 & \omega^{-\xi} \\ 1 & \omega^{-\xi} \end{bmatrix}$, $V_1 e^{\iota\xi\phi} \begin{bmatrix} 1 & \omega^{\xi} \\ \omega^{\xi} & \omega^{-\xi} \end{bmatrix}$ and $V_1 e^{\iota\xi\phi} \begin{bmatrix} 1 & 1 \\ \omega^{-\xi} & \omega^{-\xi} \end{bmatrix}$ respectively, with $\omega = e^{\iota 2\pi/3}$, $V_0 = 29$ meV, $V_1 = 21$ meV and $\phi = -0.29$ rad.

B: Band Structure Calculation

We write the Hamiltonian in the bilayer graphene basis $\{|\mathbf{q}_{m_1,m_2}, A^1\rangle, |\mathbf{q}_{m_1,m_2}, B^1\rangle, |\mathbf{q}_{m_1,m_2}, A^2\rangle, |\mathbf{q}_{m_1,m_2}, B^2\rangle\}$ where $\mathbf{q}_{m_1,m_2} = \mathbf{k} + m_1\mathbf{G}^{\mathrm{SM}}_1 + m_2\mathbf{G}^{\mathrm{SM}}_2$. $\mathbf{k}$ is wave vector in the first supermoiré Brillouin zone and $\mathbf{G}^{\mathrm{SM}}_1$ and $\mathbf{G}^{\mathrm{SM}}_2$ are the basis vectors of the supermoiré reciprocal lattice. A spherical k-space cutoff is chosen such that $|\mathbf{q}_{m_1,m_2}| \le \mathbf{q}_c$. We take the cutoff, $\mathbf{q}_c = 2|\mathbf{G}^t_1|$. This choice for the cutoff ensures the convergence of the band structure. The band structure is obtained by diagonalizing the continuum Hamiltonian across the path $\Gamma_{\mathrm{SM}} - \mathrm{K}_{\mathrm{SM}} - \mathrm{K}'_{\mathrm{SM}} - \Gamma_{\mathrm{SM}} - \mathrm{M}_{\mathrm{SM}}$ in the first supermoiré Brillouin zone. The band structures of bare bilayer graphene, bilayer graphene forming top moiré with hBN, bilayer graphene forming bottom moiré with hBN, and hBN/BLG/hBN double moiré are shown in Supplementary Figure 6(a-d).

C: Density of states (DOS) versus number density

The density of states, $g(E)$ is calculated as

$$g(E) = \frac{4}{S} \sum_{n\mathbf{k}} w_{n\mathbf{k}} \delta(E - E_{n\mathbf{k}}) \qquad \text{(Supplementary Equation 6)}$$

where S is the real space area of supermoiré first BZ. We used a uniform sampling of the supermoiré BZ of 200×200 k-points to calculate the DOS. The factor of 4 in Supplementary Equation 6 is to account for the four-fold valley and spin degeneracy.

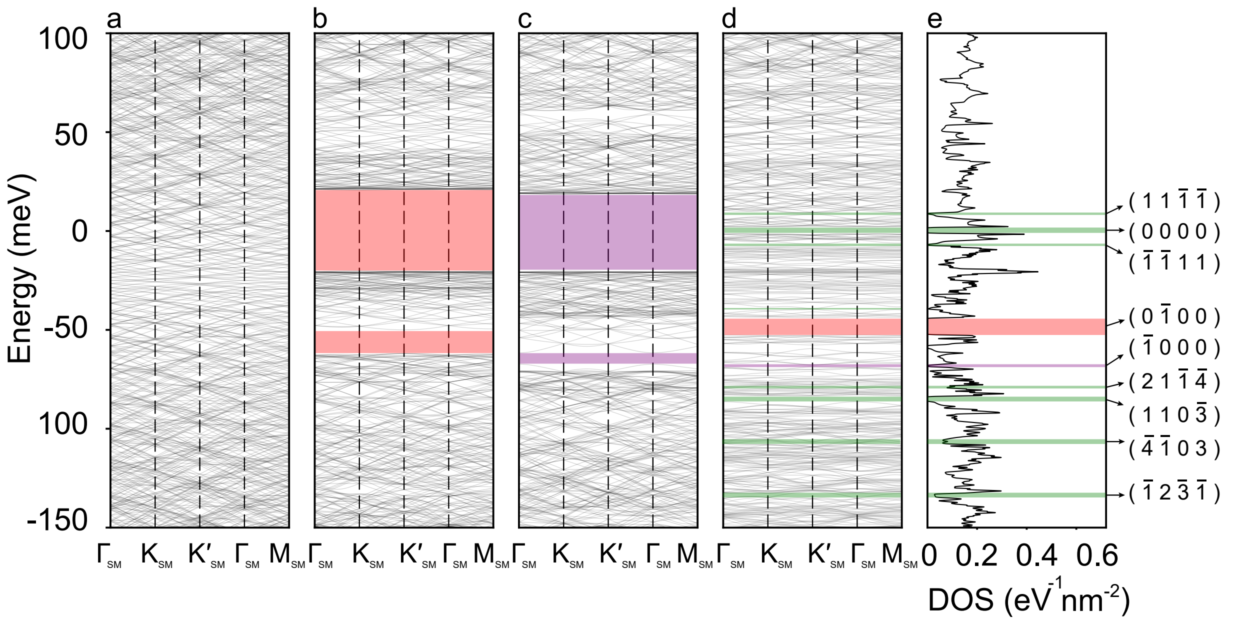

Supplementary Figure 6. Band Structure and density of states for the system with $\theta_b = 0.026°$ and $\theta_t = 0.44°$. (Plot of the band structure of bilayer graphene with (a) no moiré potential, (b) bottom moiré potential, (c) top moiré potential, and (d) double moiré potential along the path $\Gamma_{\rm SM} - {\rm K}_{\rm SM} - {\rm K}'_{\rm SM} - \Gamma_{\rm SM} - {\rm M}_{\rm SM}$ of the supermoiré BZ. (e) Plot of the DOS versus energy calculated for the supermoiré band structure in (d). The primary first-order gaps are shown in red and purple in (b), (c), (d), and (e). The secondary higher order gaps are marked in green in (d) and (e).

The delta function, $\delta(E - E_{n\mathbf{k}})$, is approximated as a Gaussian with broadening 0.17 meV corresponding to the measurement temperature of 2 K. The DOS as a function of E (with the gaps highlighted) is shown in Supplementary Figure 6(e). We also overlay the DOS of the pristine bilayer graphene on the DOS of doubly aligned BLG with hBN (Supplementary Figure 7). The DOS of pristine BLG is linear at finite E. The moiré potential results in multiple dips and gaps over the linear DOS of pristine BLG. To compare with experimental results, we convert the calculated DOS to be a function of the number density n using $n(E) = 4\sum_{n\mathbf{k}} w_{n\mathbf{k}}\Theta(E - E_{n\mathbf{k}})$, where $\Theta(E - E_{n\mathbf{k}})$ is the Heaviside step function.

SUPPLEMENTARY NOTE 5: DETERMINING THE BRAGG INDICES FOR THE GAPS

The number density corresponding to a gap is related to the areas A_1, A_2, A_3, A_4 by the set of integers m_1, m_2, m_3, m_4 as:

$$n = 4(m_1A_1 + m_2A_2 + m_3A_3 + m_4A_4)/(2\pi)^2 \qquad \text{(Supplementary Equation 7)}$$

Here areas are defined as the independent subsets of the cross products of moiré reciprocal lattice vectors. There are, thus, four unknowns in the equation. Also, the electron number density corresponding to band gaps evolves continuously with changes in the twist angle (Supplementary Figure 8). We consider four different twist angles ($\theta_t = 0.40°, 0.44°, 0.47°, 0.52°$) to find the integers for the gaps observed in $\theta_t = 0.44°$. For a commensurate system, $A_1, A_2, A_3,$ andA_4 have the highest common factor $A_{\rm SM} = \left|\mathbf{G}_1^{\rm SM} \times \mathbf{G}_2^{\rm SM}\right|$. As a result, they can be written as an integral multiple of $A_{\rm SM}$ via the relation $A_i = s_iA_{\rm SM}$ with integers s_i (i = 1, 2, 3, 4). We also characterize n_e for a gap by an integer p, such that $n_e = pA_{\rm SM}/(2\pi)^2$. p equals the number of bands from the CNP to the gap with density n_e.

Substituting the areas and number densities in terms of an integral multiple of supermoiré areas in (Supplementary Equation 7), we get the Diophantine equation [7]:

$$p = m_1s_1 + m_2s_2 + m_3s_3 + m_4s_4 \qquad \text{(Supplementary Equation 8)}$$

We construct four Diophantine equations for each number density (using the four twist angles mentioned above) [7]. The system of equations is written as $SX = P$, where S is 4×4 matrix shown as the columns s_1 to s_4 in the Supplementary Table 2. P is the column shown there as p_1 to p_7 for seven different gaps. The solution of this set of equations is unique and gives the Bragg indices, $X = (m_1, m_2, m_3, m_4)$ for each gap.

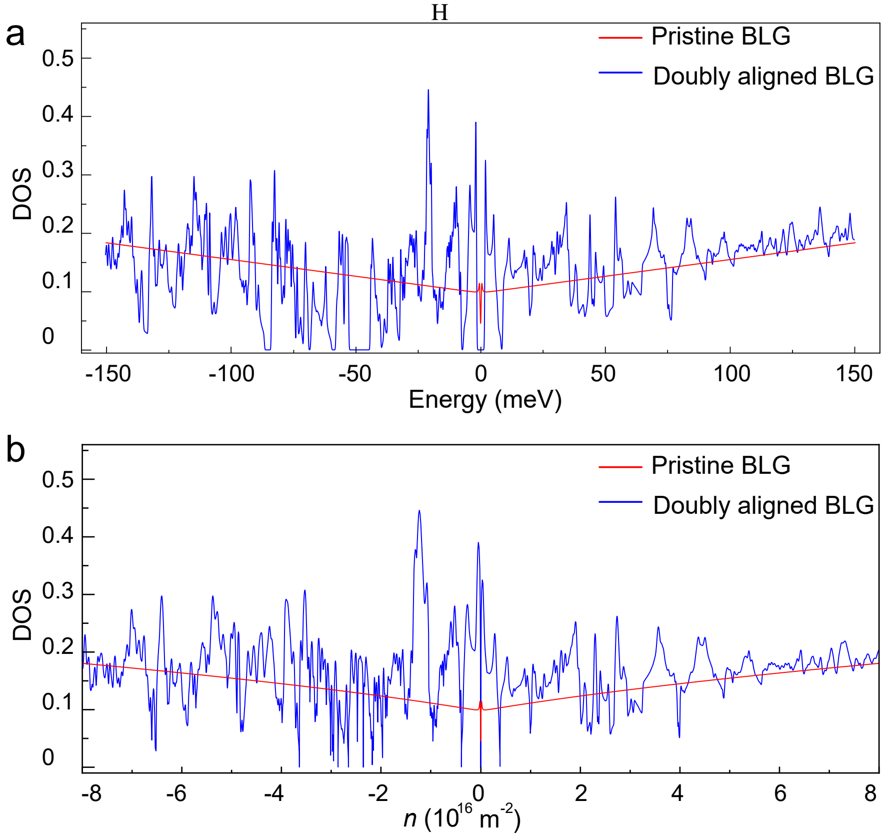

Supplementary Figure 7. Comparison of DOS. Plots comparing the calculated DOS versus (b) energy and (b) versus charge carrier density for the pristine BLG (red curve) and doubly aligned BLG with hBN with $\theta_b = 0.026°$ and $\theta_t = 0.44°$ (blue curve).

SUPPLEMENTARY NOTE 6: QUASI BRILLOUIN ZONES

Below we describe the procedure to construct the qBZ by modulating the moiré potential strength in Hamiltonian by a factor of η:

$$H_{eff} = \begin{bmatrix} H_G + \eta V^b_{hBN} & U^\dagger_{BLG} \\ U_{BLG} & H_G + \eta V^t_{hBN} \end{bmatrix} \qquad \text{(Supplementary Equation 9)}$$

We plot the unfolded the band structure across the path Γ_{SM} to a point P in $k_x - k_y$ plane for $\eta = 0.0, 0.1, 0.2, 0.5, 0.8$ & 1.0 (Supplementary Figure 9). At $\eta = 0$, we see the bilayer graphene parabolic dispersion in the unfolded band structure, and for $\eta = 0.1, 0.2, 0.5, 0.8, 1.0$, we see gaps appearing in the dispersion with the increasing η. We trace the gap-opening points along this path. We repeat the procedure to mark the gap opening points on the whole $k_x - k_y$ plane. The qBZs thus constructed are shown in Fig.(3) of the main text.

θ_t	s_1	s_2	s_3	s_4	p_1	p_2	p_3	p_4	p_5	p_6	p_7	$A_{\mathrm{SM}}(\times 10^{-2} nm^{-2})$
0.40	61	52	42	64	-7	-52	-61	-72	-79	-104	-147	0.441
0.44	93	79	61	98	-13	-79	-93	-109	-122	-157	-216	0.297
0.47	19	16	12	20	-3	-16	-19	-22	-25	-32	-43	1.484
0.52	39	31	23	40	-7	-31	-39	-43	-50	-67	-86	0.7491

Supplementary Table 2. Tabulating the Diophantine equations for the gaps of 4 different angles (θ_t) as the augmented matrix form of the system of equations, (S|P), where S is the 4×4 matrix from column $(2 - 5)$ and the P in column $(6 - 11)$ are the integer equivalents of the number densities for 7 gaps. The last column (A_{SM}) is the reciprocal space area of supermoiré.

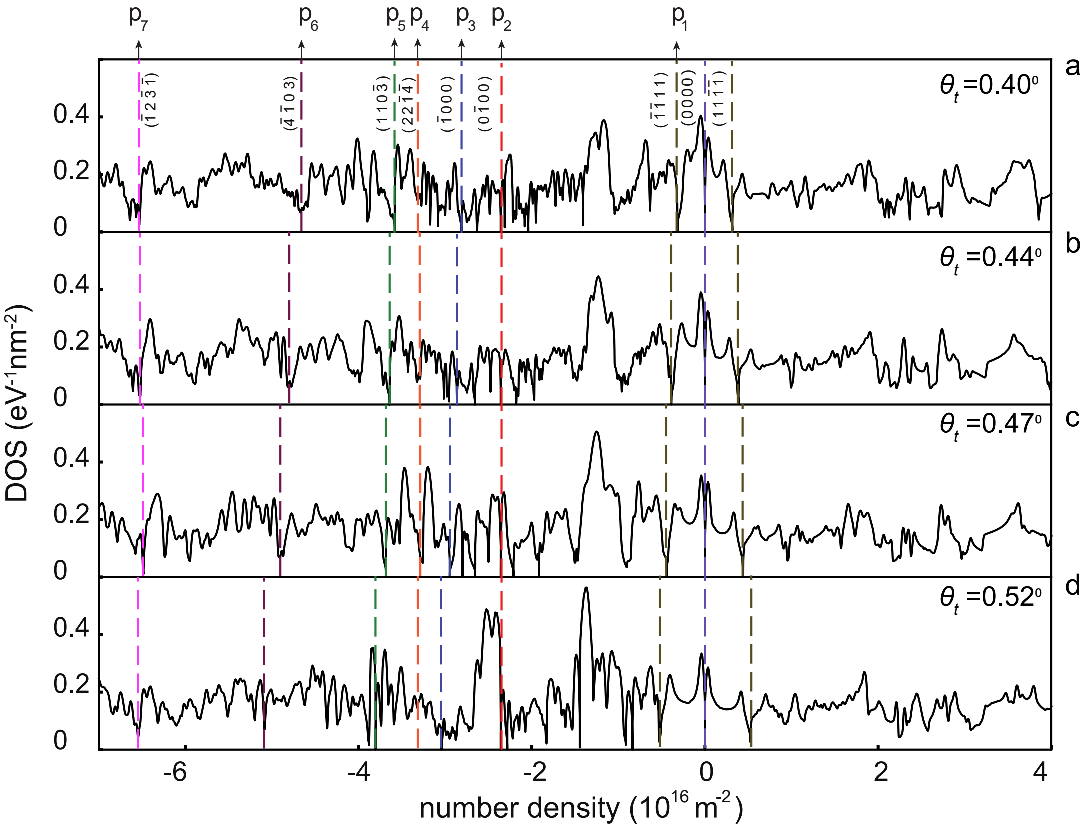

Supplementary Figure 8. DOS versus n for 4 different angles. (a)-(d) shows the DOS versus n for $\theta_t = 0.40°,\ 0.44°,\ 0.47°,\ 0.52°$ respectively. The positions of densities corresponding to the gaps, with Bragg indices marked, are shown with dotted lines.

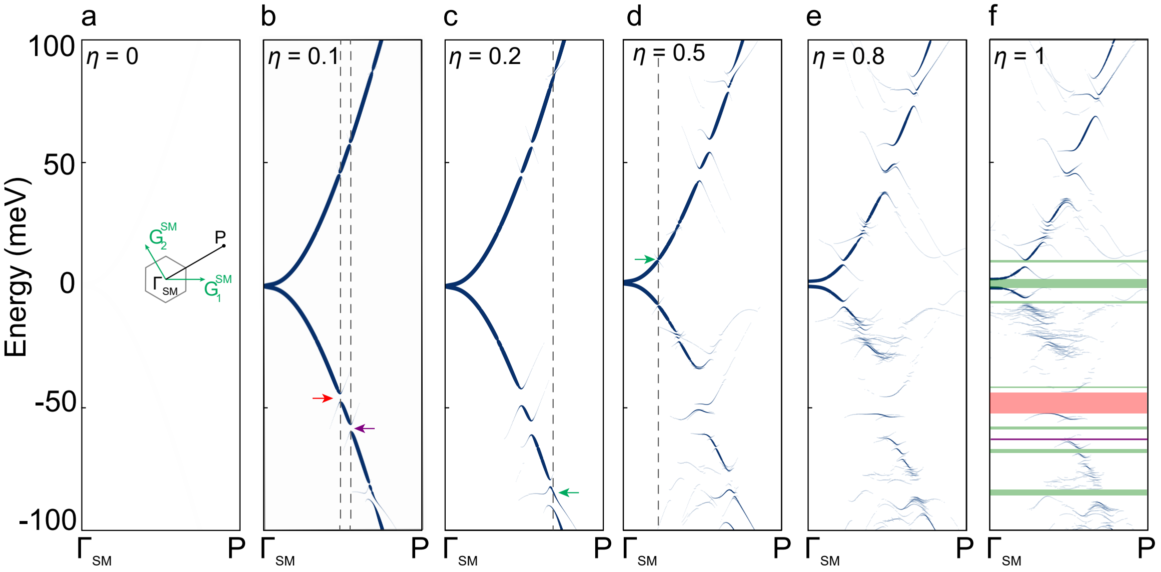

Supplementary Figure 9. Band structure for a range of moiré potential strengths parameterised by η. Band structure across the path $\Gamma_{\rm SM}$ to P unfolded to the unit cell of bilayer graphene. (a) The parabolic-like bands of bilayer graphene with no moiré potential. (b), (c), (d), (e), and (f) show the band structure with η varying from 0.1 to 1.0. The calculated spectral weights are shown as purple dots. The gap opening points can be traced back at different η. Vertical dashed lines represent the **k** point at which the gap opens along this path in the weak potential limit.

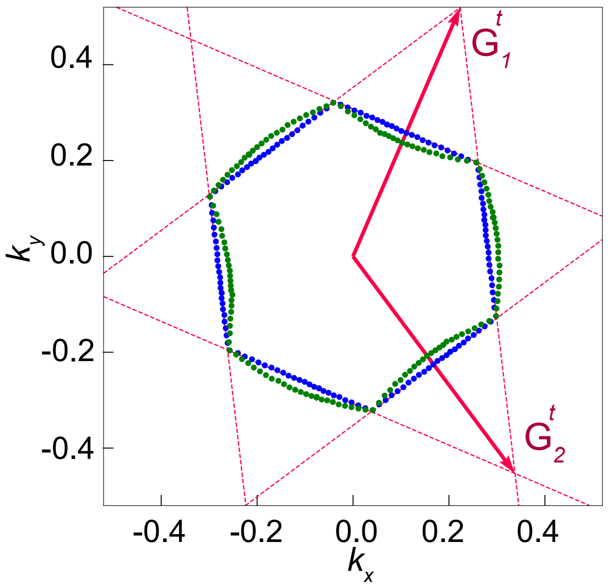

Supplementary Figure 10. qBZs for SLG and BLG aligned with hBN. Brillouin Zones constructed by unfolding the primary moiré gaps for twist angle of $\theta_t = 0.44°$. The blue (green) filled circles show the BZ of single-layer graphene/hBN (bilayer graphene/hBN) moiré. The area of each zone is equal to the corresponding number density at which the gap is observed.

SUPPLEMENTARY NOTE 7: COMPARISON OF QBZ IN SLG AND BLG

Some of the qBZs constructed for the bilayer graphene doubly aligned with hBN are distorted hexagons (see Fig.3, main text). To understand their origin, we compare the qBZ for single moiré formed between single-layer graphene (SLG) and hBN with that formed between bilayer graphene and hBN. In both cases, the angular misalignment between the graphene and the hBN was set to be $\theta_t = 0.44°$. The resultant qBZ are plotted in Supplementary Figure 10. The qBZ in the case of SLG is perfectly hexagonal; this can be understood by considering the circular iso-energy contours in the graphene dispersion. We translate the contours by vectors, $\mathbf{G}_i^t$ ($i = 1$ to 6). The intersection of the iso-energy contours gives the hexagonal qBZ. For the BLG, on the contrary, one has constant energy contours with triangular symmetry due to the trigonal warping term in the Hamiltonian. A similar intersection procedure results in distorted hexagons. (Supplementary Figure 11).

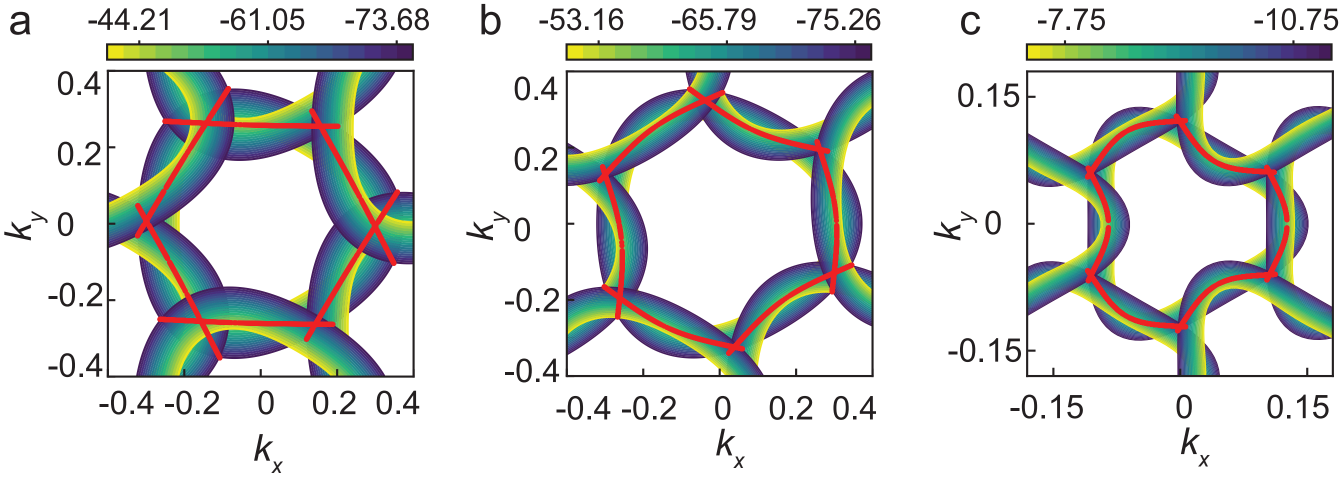

Supplementary Figure 11. BZ construction using intersection of iso-energy contours. (a), (b), and (c) show the BZ constructed by the intersection of iso-energy contours placed at $\mathbf{G}_i^b, \mathbf{G}_i^t, \mathbf{G}_i^b - \mathbf{G}_i^t$ with the dispersion centred at (0,0) in $k_x - k_y$ plane. Here $i = 1$ to 6. k_x and k_x are in nm^{-1} and colorbars in meV. These zones agree with those constructed via the unfolding procedure in Fig.(3) of the main manuscript.

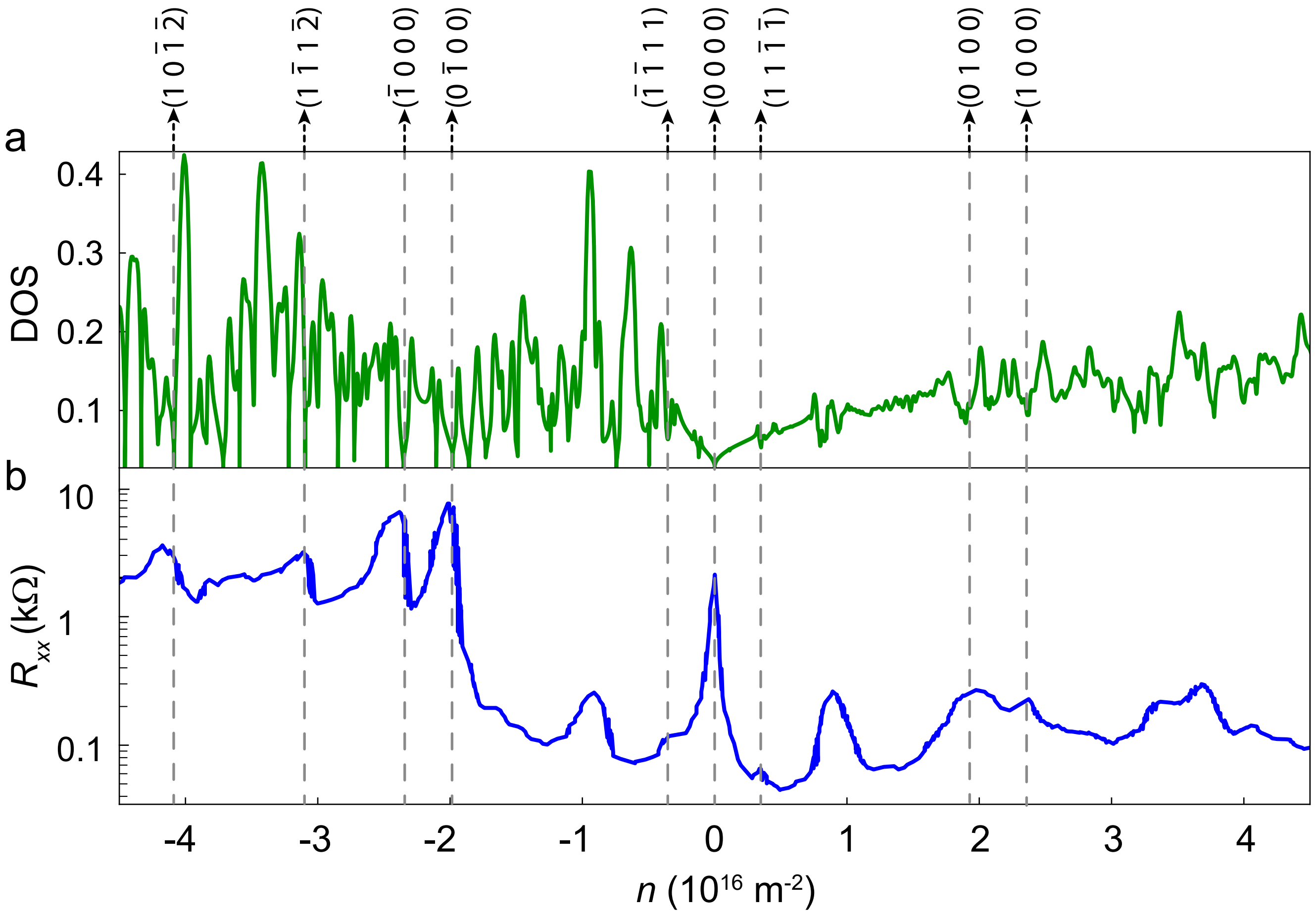

Supplementary Figure 12. Experimentally measured and theoretically calculated Bragg gaps (a) Plot of the density of states (DOS) versus n for twist angle of $\theta_b = 0°$ and $\theta_t = 0.407°$. (b) Plot of Longitudinal resistance R_{xx} versus n from Ref. [5].The vertical dashed lines mark the major dips in the DOS and corresponding peaks in the resistance curve. Corresponding Bragg indices are marked on the top.

SUPPLEMENTARY NOTE 8: ANALYSIS OF PREVIOUSLY PUBLISHED DATA ON SLG SUPERMOIRÉ

In a system of graphene aligned with a single hBN, a single moiré periodicity is typically generated, resulting in one secondary Dirac point on both the electron and hole side. However, when graphene is doubly aligned with hBN, it produces multiple gaps in addition to the primary gaps of the top and bottom moiré. This is due to the interference between the two moiré potentials. These gaps appear as peaks in the resistance versus carrier density curve during electronic transport measurements, as demonstrated in bilayer graphene data (shown in the main text Fig.(1)) and previous studies from other groups in single layer graphene(SLG) [5].

The data published in Ref.[5] are reproduced in Supplementary Figure 12. This pioneering study used a first-order approach to explain the origin of multiple peaks in the system. However, some prominent peaks, for example, those at $\mathrm{n} \approx \pm 3.10 \times 10^{16}\ \mathrm{m}^{-2}$ and $\mathrm{n} \approx \pm 4.1 \times 10^{16}\ \mathrm{m}^{-2}$, remained unexplained. We show that the origin of these peaks can be understood under a generalized continuum model approach. The effective continuum Hamiltonian for hBN/SLG/hBN is:

$$H = H_G + V^b_{hBN} + V^t_{hBN} \qquad \text{(Supplementary Equation 10)}$$

We use the lattice constants for graphene and hBN as 0.2464 and 0.2504 nm, respectively, to have the lattice mismatch of 1.6% (as mentioned by the authors of Ref.[5]). Supplementary Figure 12 shows the theoretical density of states for the given twist angles $\theta_b = 0°$ and $\theta_t = 0.407°$.The areas A_i for the experimentally obtained twist angle are 0.233, 0.196, 0.147 and 0.245 nm^{-2}. The gaps at $n = -1.98 \times 10^{16}\ \mathrm{m}^{-2}$ and $n = -2.36 \times 10^{16}\ \mathrm{m}^{-2}$ corresponds to Bragg indices $(0, \bar{1}, 0, 0)$ and $(\bar{1}, 0, 0, 0)$ respectively. The peak in R_{xx} at at $n = -0.37 \times 10^{16}\ \mathrm{m}^{-2}$ corresponds to Bragg indices $(\bar{1}, \bar{1}, 1, 1)$ and is identified to be the supermoiré gap. Peaks that went unexplained in the original publication at $n = -3.10 \times 10^{16}\ \mathrm{m}^{-2}$ and $\mathrm{n} = -4.1 \times 10^{16}\ \mathrm{m}^{-2}$

θ_t	s_1	s_2	s_3	s_4	p_1	p_2	p_3	p_4	p_5	$A_{\rm SM}(\times 10^{-2}\ {\rm nm}^{-2})$
0.33	28	25	20	30	-3	-25	-28	-37	-52	0.784
0.36	31	27	21	33	-4	-27	-31	-41	-56	0.726
0.41	19	16	12	20	-3	-16	-19	-25	-33	1.225
0.47	61	49	35	63	-12	-49	-61	-79	-100	0.4
0.54	4	3	2	4	-1	-3	-4	-5	-6	6.542

Supplementary Table 3. Tabulating the Diophantine equations for the gaps of 5 different angles (θ_t) as the augmented matrix form of the system of equations, (S|P), where S is the 5×4 matrix from column $(2-5)$ and the P in column $(6-11)$ are the integer equivalents of the number densities for 5 gaps with Bragg indices $(\bar{1},\bar{1},1,1)$, $(0,\bar{1},0,0)$, $(\bar{1},0,0,0)$, $(1,\bar{1},1,\bar{2})$ and $(1,0,\bar{1},\bar{2})$ respectively. The last column ($A_{\rm SM}$) is the area of the reciprocal space supermoiré cell.

can now be understood to arise due to the noticeable zeros in the calculated density of states and correspond to the Bragg indices $(1,\bar{1},1,\bar{2})$ and $(1,0,\bar{1},\bar{2})$ respectively.

SUPPLEMENTARY NOTE 9: LANDAU FAN DIAGRAM

Supplementary Figure 13 shows the Quantum Hall Landau fan diagram G_{xx} of the device, measured with respect to carrier density n and magnetic field B at a temperature of $T = 2$ K. The emergence of Landau levels can be identified as conductance minima, fanning out from the primary and the secondary Dirac points. In Supplementary Figure 13(b), specific features from Supplementary Figure 13(a) are highlighted, Landau levels with filling value ($\nu = \pm 4$m, where m =integer), which emerge from the charge neutrality point are depicted with red dotted lines. These Quantum Hall states correspond to states of conventional bilayer graphene, further affirming the presence of the bilayer graphene in the system. Moreover, the fan diagram exhibits weak features of horizontal conductance maxima streaks, represented by cyan-coloured arrow lines. These lines are recognized as Brown-Zak lines, originating from the recurring Bloch states in the superlattice.

SUPPLEMENTARY NOTE 10: ABSENCE OF SUPERMOIRÉ PEAK IN RESISTANCE MEASUREMENTS

We do not see the superlattice peak in the resistance versus carrier density response; this is in agreement with the previous study on the bilayer graphene supermoiré system [8]. There can be two possible reasons for not observing the supermoiré peak in resistance:

1. We estimate the magnitude of the supermoiré gap to be $\Delta_s \approx 0.63$ meV – it is unlikely that such a small gap will give rise to a substantial resistance peak.

2. From the measured Brown-Zak oscillations, we estimate the carrier density at which the superlattice gap should open to be $n_s = 0.39 \times 10^{16}\ {\rm m}^{-2}$. We do not see a clear peak at this carrier density, but we do find that the FWHM of the charge neutrality point is greatly increased for the supermoiré as compared to that of the single moiré device (Supplementary Figure 3). We believe that the supermoiré peak, if it appeared in resistance measurements, would be subsumed by the peak of the primary Dirac point.

SUPPLEMENTARY NOTE 11: COMPARING HBN DOUBLE MOIRÉ SYSTEM OF SLG AND BLG

Supplementary Figure 14 shows the calculated DOS for both the SLG/hBN double moiré system and BLG/hBN double moiré system at the same twist angles ($\theta_b = 0.03°$ and $\theta_t = 0.44°$) using the continuum model. It shows that most of the prominent dips observed in the BLG double moiré system convert to sizable band gaps in the SLG double moiré system. We also find that the size of the band gaps E_g is consistently larger for SLG double moiré, in comparison to BLG double moiré (see Supplementary Table 4). The exact reasoning behind this is unclear and requires further studies.

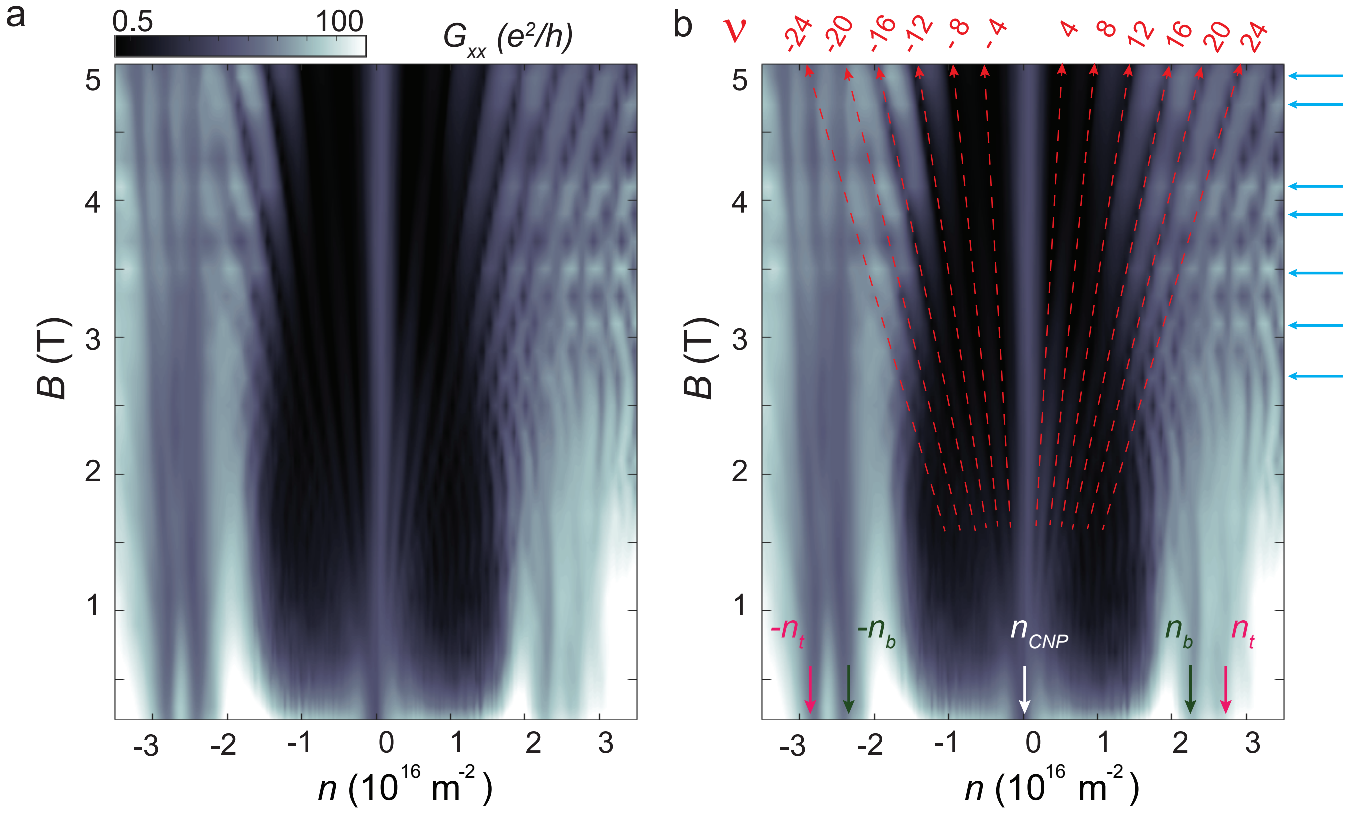

Supplementary Figure 13. Landau fan diagram of the double moiré device. (a) Raw data of the Landau-fan diagram $G_{xx}(n, B)$, showing the emergence of Landau levels from the primary and the secondary Dirac points. (b) Quantum Hall effect states corresponding to conventional bilayer graphene are marked by red dotted lines with the corresponding Landau filling value ($\nu = \pm 4$m, where m =integer). The white line marks the charge neutrality point. Magenta and green lines indicate the secondary Dirac points emerging from top and bottom moiré respectively, with carrier density (moiré wavelength) $n_t = \pm 2.80 \times 10^{16}$ m^{-2} ($\lambda_t = 12.84$ nm) and $n_b = \pm 2.36 \times 10^{16}$ m^{-2} ($\lambda_b = 13.97$ nm), respectively. The cyan horizontal arrows mark the weak Brown-Zak features. The measurements were done at $T = 2$ K.

n ($\times 10^{16}$ m^{-2})	E_g for BLG (meV)	E_g for SLG (meV)	quantum numbers
-0.39	0.63	1.06	$\bar{1}\bar{1}11$
-2.36	7.70	8.85	$0\bar{1}00$
-2.80	0.25	4.17	$\bar{1}000$
-4.8	-	1.98	$\bar{4}\bar{1}03$

Supplementary Table 4. Gap size comparison between BLG and SLG double moiré for twist angles $\theta_b = 0.03°$ and $\theta_t = 0.44°$.

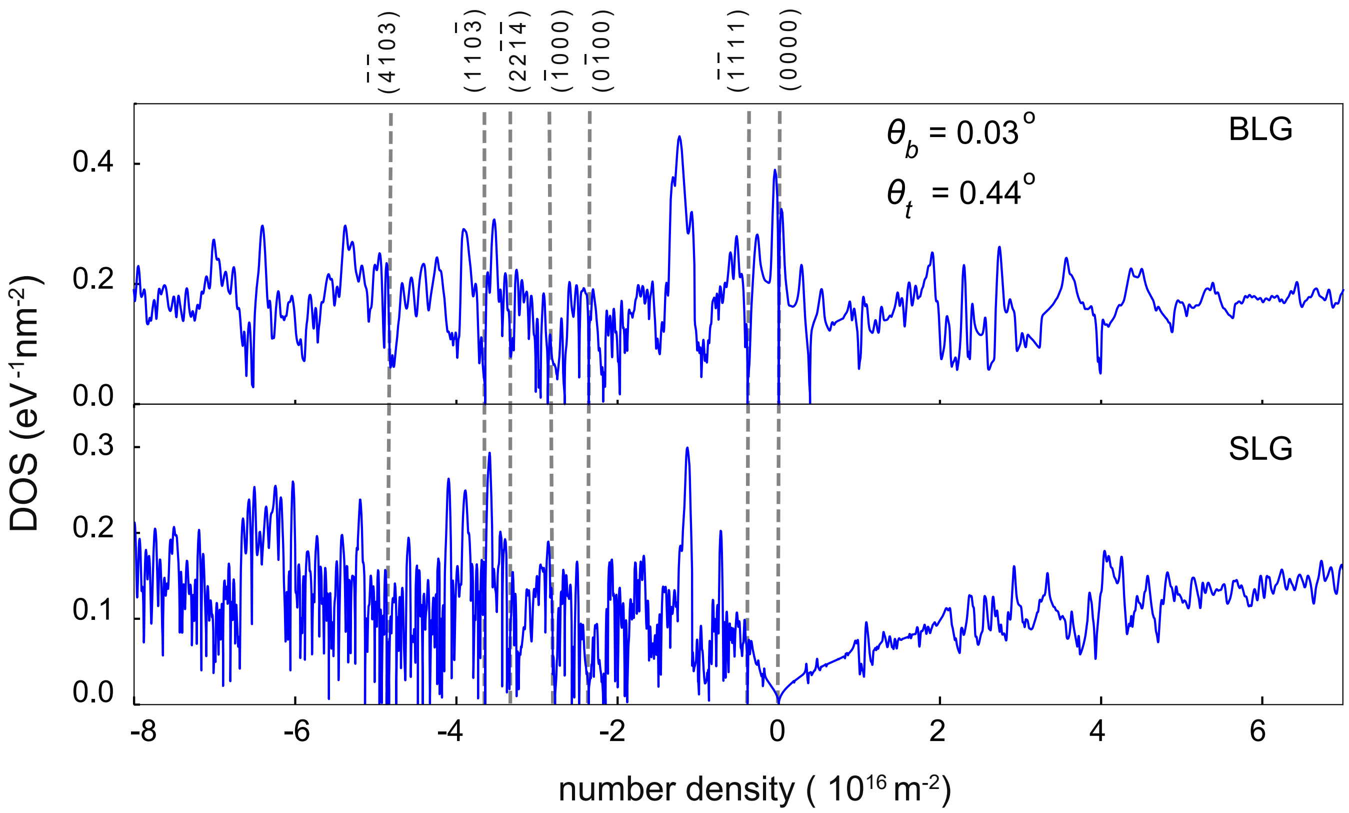

Supplementary Figure 14. Calculated DOS for the (a) SLG/hBN double moiré system and (b) BLG/hBN double moiré system at the same twist angles ($\theta_b = 0.03°$ and $\theta_t = 0.44°$) using the continuum model. The vertical dash line marks the higher order Bragg gaps with the corresponding quantum numbers.

SUPPLEMENTARY REFERENCES

* mjain@iisc.ac.in
† aveek@iisc.ac.in

[1] L. Wang, I. Meric, P. Huang, Q. Gao, Y. Gao, H. Tran, T. Taniguchi, K. Watanabe, L. Campos, D. Muller, et al., One-dimensional electrical contact to a two-dimensional material, Science **342**, 614 (2013).
[2] P. Moon and M. Koshino, Electronic properties of graphene/hexagonal-boron-nitride moiré superlattice, Phys. Rev. B **90**, 155406 (2014).
[3] B. Hunt, J. D. Sanchez-Yamagishi, A. F. Young, M. Yankowitz, B. J. LeRoy, K. Watanabe, T. Taniguchi, P. Moon, M. Koshino, P. Jarillo-Herrero, and R. C. Ashoori, Massive dirac fermions and hofstadter butterfly in a van der waals heterostructure, Science **340**, 1427 (2013).
[4] L. Wang, S. Zihlmann, M.-H. Liu, P. Makk, K. Watanabe, T. Taniguchi, A. Baumgartner, and C. Schönenberger, New generation of moiré superlattices in doubly aligned hbn/graphene/hbn heterostructures, Nano Letters **19**, 2371 (2019).
[5] Z. Wang, Y. B. Wang, J. Yin, E. Tóvári, Y. Yang, L. Lin, M. Holwill, J. Birkbeck, D. J. Perello, S. Xu, J. Zultak, R. V. Gorbachev, A. V. Kretinin, T. Taniguchi, K. Watanabe, S. V. Morozov, M. Anđelković, S. P. Milovanović, L. Covaci, F. M. Peeters, A. Mishchenko, A. K. Geim, K. S. Novoselov, V. I. Falko, A. Knothe, and C. R. Woods, Composite super-moiré lattices in double-aligned graphene heterostructures, Science Advances **5**, eaay8897 (2019).
[6] R. Smeyers, M. V. Milošević, and L. Covaci, Strong gate-tunability of flat bands in bilayer graphene due to moiré encapsulation between hbn monolayers, Nanoscale **15**, 4561 (2023).
[7] H. Oka and M. Koshino, Fractal energy gaps and topological invariants in hbn/graphene/hbn double moiré systems, Phys. Rev. B **104**, 035306 (2021).
[8] M. Kuiri, S. K. Srivastav, S. Ray, K. Watanabe, T. Taniguchi, T. Das, and A. Das, Enhanced electron-phonon coupling in doubly aligned hexagonal boron nitride bilayer graphene heterostructure, Phys. Rev. B **103**, 115419 (2021).
